# Supplementary material for: An Electrochemical Immunosensor for the Determination of Procalcitonin Using the Gold-Graphene Interdigitated Electrode
Source: Biosensors (Basel). 2022 Sep 20;12(10):771. doi: 10.3390/bios12100771 (PMC9599768; doi:10.3390/bios12100771)
Supplement: Supplementary file 1 [file biosensors-12-00771-s001.zip › biosensors-1928966-supplementary.pdf]

biosensors

MDPI

Supplementary material

An electrochemical immunosensor for the determination of procalcitonin using the gold-graphene interdigitated electrode

Mahmoud Amouzadeh Tabrizi* and Pablo Acedo

Electronic Technology Department, Universidad Carlos III de Madrid, Leganés, Spain

*Correspondence: : mamouzad@ing.uc3m.es, mahmoud.tabrizi@gmail.com (M.A.Tabrizi)

A

B

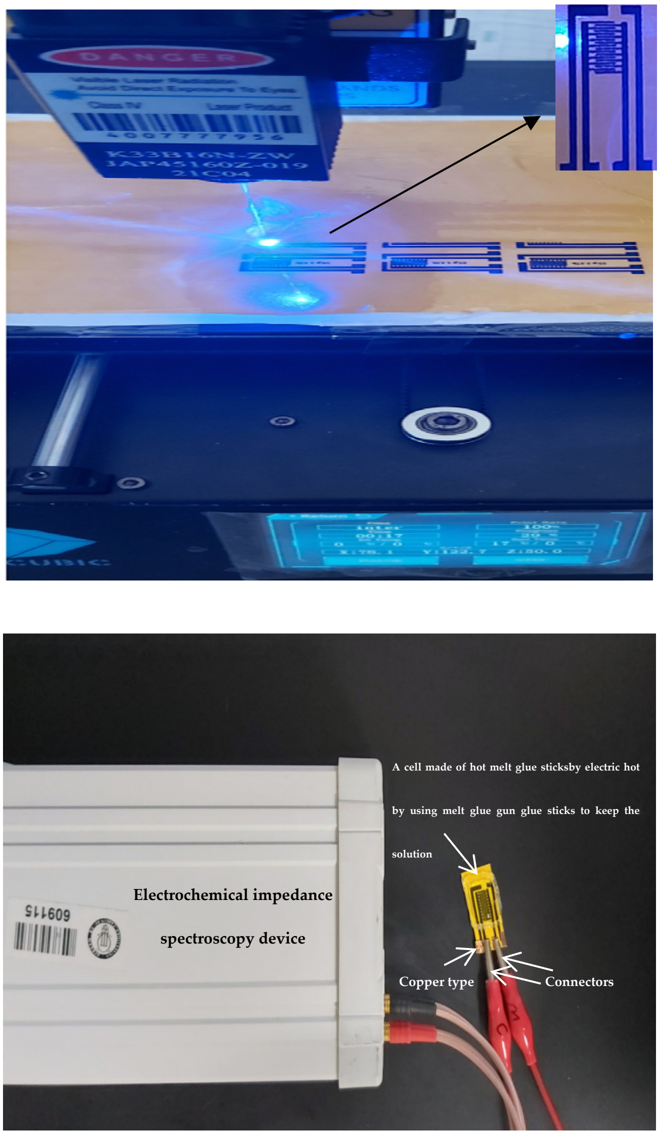

Figure S1. (A) Photo image of the process of the fabrication of the LDGE using a laser diode. (B) Photo image of the electrochemical cell.

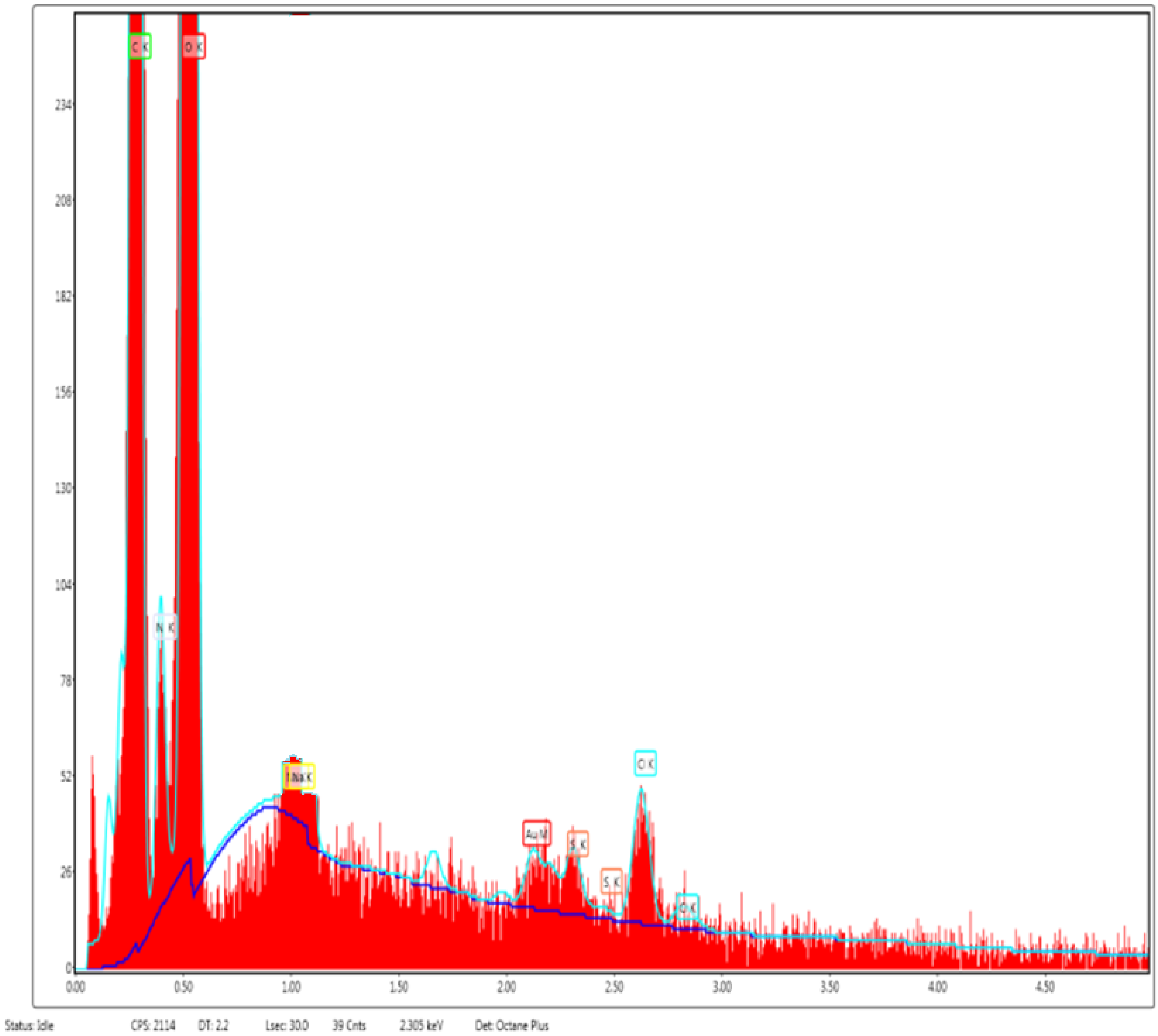

Figure S2. EDX of the IDGE/Au_{nano}/3-MPA/Anti-PTC/BSA.

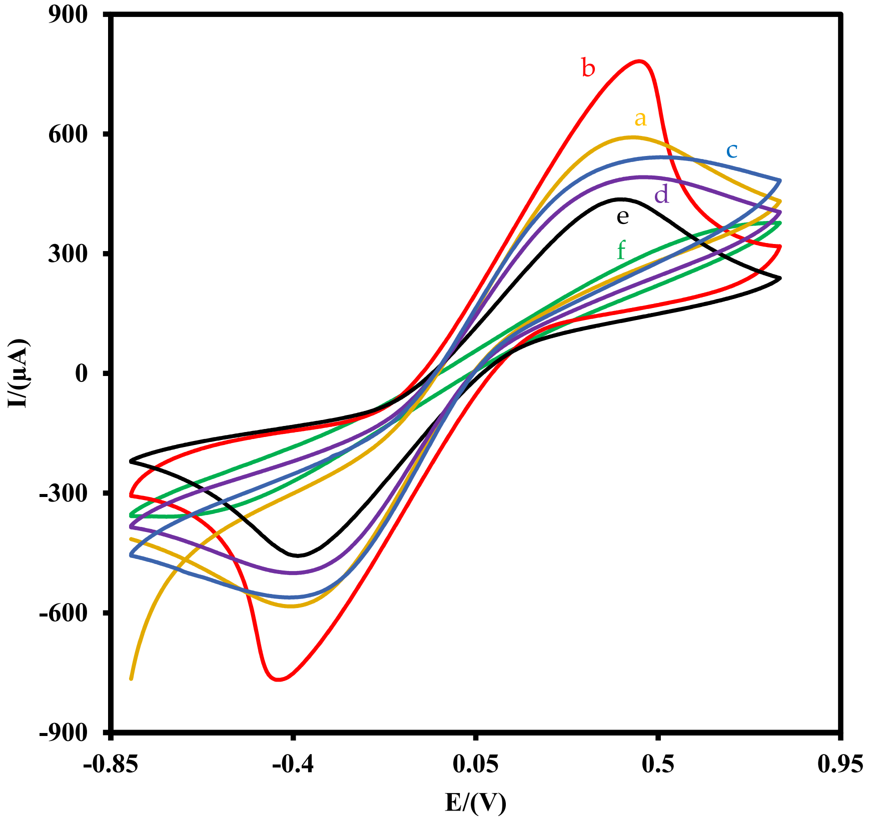

Figure S3. Single mode CVs of the IDGE (a), IDGE/Au$_{nano}$ (b), IDGE/Au$_{nano}$/3-MPA (c), IDGE/Au$_{nano}$/3-MPA/Anti-PCT (d), IDGE/Au$_{nano}$/3-MPA/Anti-PCT/BSA (e), IDGE/Au$_{nano}$/3-MPA/Anti-PCT/BSA/PCT (f) in 5 mM $Fe(CN)_6^{3-/4-}$ couple (1:1) and 0.1 M PBS at scan rate of 0.05 υ/s.

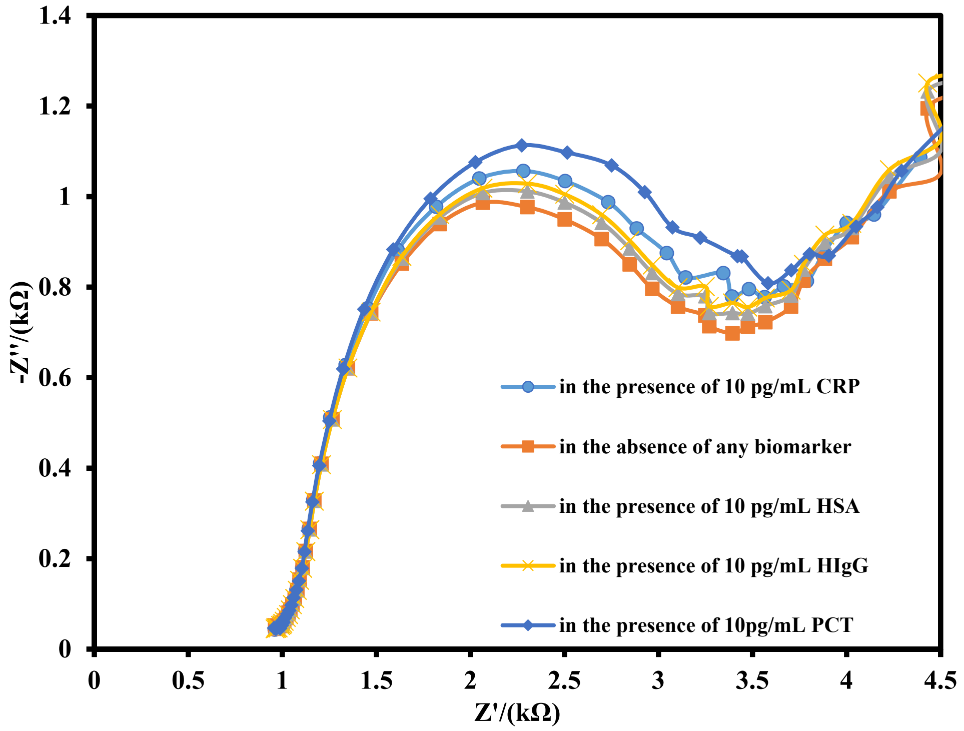

Figure S4. EIS response of IDGE/Au_{nano}/3-MPA/Anti-PCT/BSA in the absence of any biomarkers and the presence of biomarkers. EIS measurements were done in 5 mM $Fe(CN)_6^{3-/4-}$ couple (1:1) and 0.1 M PBS. The value of R_{et} in the absence of any biomarker was 2744 Ω, and in the presence of 10 pg/mL PCT, CRP, HIgG, and HSA were 2993 Ω, 2790 Ω, 2775 Ω, and 2744 Ω, respectively. Nyquist diagrams. R_s: Solution resistance, R_{et}: Electron transfer resistance, C_{dl}: Double layer capacitance, Z_w: Warburg impedance. AC amplitude voltage was 10 mV, DC voltage was 0 V, and frequency range was 100000 Hz-0.1 Hz.
